# Cold water immersion in recovery following a single bout resistance exercise suppresses mechanisms of miRNA nuclear export and maturation

**DOI:** 10.14814/phy2.15784

**Published:** 2023-08-07

**Authors:** Randall F. D'Souza, Vandre C. Figueiredo, James F. Markworth, Nina Zeng, Christopher P. Hedges, Llion A. Roberts, Truls Raastad, Jeff S. Coombes, Jonathan M. Peake, Cameron J. Mitchell, David Cameron‐Smith

**Affiliations:** ^1^ Liggins Institute The University of Auckland Auckland New Zealand; ^2^ Discipline of Nutrition The University of Auckland Auckland New Zealand; ^3^ Maurice Wilkins Centre for Molecular Biodiscovery The University of Auckland Auckland New Zealand; ^4^ Department of Biological Sciences Oakland University Rochester Michigan USA; ^5^ Department of Animal Science Purdue University West Lafayette Indiana USA; ^6^ Department of Physiology The University of Auckland Auckland New Zealand; ^7^ School of Human Movement and Nutrition Sciences University of Queensland Brisbane Queensland Australia; ^8^ Sports Performance Innovation and Knowledge Excellence Queensland Academy of Sport Brisbane Queensland Australia; ^9^ School of Health Sciences and Social Work Griffith University Gold Coast Queensland Australia; ^10^ Department of Physical Performance Norwegian School of Sport Sciences Oslo Norway; ^11^ School of Biomedical Sciences Queensland University of Technology Brisbane Queensland Australia; ^12^ School of Kinesiology University of British Colombia Vancouver British Columbia Canada; ^13^ College of Engineering, Science and Environment University of Newcastle Callaghan New South Wales Australia

**Keywords:** cold water immersion, microRNA export and maturation, resistance exercise

## Abstract

Cold water immersion (CWI) following intense exercise is a common athletic recovery practice. However, CWI impacts muscle adaptations to exercise training, with attenuated muscle hypertrophy and increased angiogenesis. Tissue temperature modulates the abundance of specific miRNA species and thus CWI may affect muscle adaptations via modulating miRNA expression following a bout of exercise. The current study focused on the regulatory mechanisms involved in cleavage and nuclear export of mature miRNA, including *DROSHA*, *EXPORTIN‐5*, and *DICER*. Muscle biopsies were obtained from the *vastus lateralis* of young males (*n* = 9) at rest and at 2, 4, and 48 h of recovery from an acute bout of resistance exercise, followed by either 10 min of active recovery (ACT) at ambient temperature or CWI at 10°C. The abundance of key miRNA species in the regulation of intracellular anabolic signaling (miR‐1 and miR‐133a) and angiogenesis (miR‐15a and miR‐126) were measured, along with several gene targets implicated in satellite cell dynamics (NCAM and PAX7) and angiogenesis (VEGF and SPRED‐1). When compared to ACT, CWI suppressed mRNA expression of *DROSHA* (24 h *p* = 0.025 and 48 h *p* = 0.017), *EXPORTIN‐5* (24 h *p* = 0.008), and *DICER* (24 h *p* = 0.0034). Of the analyzed miRNA species, miR‐133a (24 h *p* < 0.001 and 48 h *p* = 0.007) and miR‐126 (24 h *p* < 0.001 and 48 h *p* < 0.001) remained elevated at 24 h post‐exercise in the CWI trial only. Potential gene targets of these miRNA, however, did not differ between trials. CWI may therefore impact miRNA abundance in skeletal muscle, although the precise physiological relevance needs further investigation.

## 1 INTRODUCTION

Cryotherapy, most performed using cold water immersion (CWI), is widely employed during the recovery phase post‐exercise or high intensity sport events. CWI has been reported to attenuate muscle soreness and increase the speed of recovery of muscular function following exercise (Fonseca et al., 2016; Moore et al., 2022; Siqueira et al., 2018). While CWI following intense resistance exercise may be beneficial for sub‐maximal aerobic function (Roberts et al., 2014), there is also evidence for reduced post‐exercise rates of muscle protein synthesis rates (Fuchs et al., 2020) and blunted resistance training‐induced muscle hypertrophy and muscular strength (Roberts et al., 2015). CWI use with resistance training also shifts muscle cell adaptations toward a relatively slow twitch phenotype, with increased capillary formation (D'Souza, Zeng, Markworth, et al., 2018). Therefore, CWI exerts pleiotropic effects on the adaptive cellular and molecular responses within the musculature following a bout of exercise.

Given the diversity of molecular mechanisms regulated by CWI, one unexplored potential cellular mechanism influenced by the transient muscle hypothermia is microRNAs (miRNAs). miRNAs are well‐established to be important components of the molecular events necessary for muscle phenotype adaptation to physical activity and exercise training (D'Souza, Bjørnsen, et al., 2017; D'Souza, Markworth, et al., 2017; D'Souza, Zeng, Figueiredo, et al., 2018; D'Souza, Zeng, Markworth, et al., 2018; D'Souza, Zeng, Markworth, et al., 2019; D'Souza, Zeng, Poppitt, et al., 2019; Margolis et al., 2017; Margolis & Rivas, 2018; Mitchell et al., 2018; Zacharewicz et al., 2014; Zeng et al., 2018). More than 2000 human miRNA species have been reported (Kozomara & Griffiths‐Jones, 2014). miRNAs function to “fine‐tune” protein expression, by either binding to and promoting mRNA degradation, or by inhibiting the translational complex (Sevignani et al., 2006; Ying & Lin, 2004). Cellular abundance of miRNA species is initially regulated by transcriptional activation. miRNA species are firstly transcribed as long primary‐miRNAs (pri‐miRNA) that then undergo cleavage by an enzymatic complex that includes DROSHA, a ribonuclease III endonuclease, resulting in ~70 nucleotide long precursor miRNA (pre‐miRNA). The generated pre‐miRNAs are subsequently exported from the nucleus by EXPORTIN‐5, and then undergo final cleavage by another related ribonuclease III endonuclease, DICER, generating the mature ~19–22 nucleotide miRNA (Drummond et al., 2008). Therefore, miRNA abundance is controlled by multiple mechanisms, with miRNA cleavage and trafficking being key steps. Consistent with the role of miRNA in mediating aspects of the molecular adaptations to exercise, and the adaptive regulation of transcript maturation and traffic, previous studies have reported that the gene expression of *DROSHA*, *EXPORTIN‐5*, and *DICER* are all upregulated following resistance exercise (Garner et al., 2020; Russell et al., 2013).

Transient hypothermia and hyperthermia both exert a pronounced influence on miRNA abundances and their transcriptional activity in multiple tissue and cell types (Chen et al., 2018; Fukuoka et al., 2014; Liu et al., 2017; Umehara et al., 2020). More specifically to exercise, in long‐distance runners who experienced hypothermia there was differential regulation of muscle miRNA species compared to runners who did not experience hypothermia (Devasthanam & Tomasi, 2017; Umehara et al., 2020). Further, cell culture studies have identified that the DICER protein is highly sensitive to both hyperthermia and hypothermia (Pilotte et al., 2011). Collectively, these findings provide a hypothetical basis for investigation of the impact of CWI on the molecular mechanisms of miRNA regulation (DROSHA, EXPORTIN‐5, and DICER) and expression of specific miRNA species related to exercise adaptations in response to CWI during exercise recovery (Peake et al., 2020). On the basis of prior cell culture studies (Devasthanam & Tomasi, 2017), we hypothesized that CWI would elevate muscle DICER expression following a single bout of resistance exercise. Further analysis was conducted to quantify various miRNA species previously implicated in the adaptive response to muscle loading including intracellular anabolic signaling (miR‐1 and miR‐133a; McCarthy & Esser, 2007) and angiogenesis (miR‐15a and miR‐126a; Fish et al., 2008; Sun et al., 2013; Wang et al., 2016; Yin et al., 2012). Finally, analysis was made of downstream mRNA and protein targets of these miRNAs involved in myogenesis (neural cell adhesion molecule; *NCAM* and paired protein box 7; *PAX7*) (Chen et al., 2010; Gagan et al., 2012; Harding & Velleman, 2016; Ivey et al., 2008; Yin et al., 2020) and angiogenesis (vascular endothelial growth factor; *VEGF*, and Sprouty‐related EVH1 domain‐containing protein 1; *SPRED‐1*) (Cordes & Srivastava, 2009; Dang et al., 2013; Henn et al., 2019; Wang & Olson, 2009).

## 2 METHODS

Nine physically active men (22.1 ± 2.2 years old), with a history of at least 12 months experience in strength training completed a bout of single‐leg strength exercise on two separate occasions using opposite legs, separated by at least 1 week (Roberts et al., 2015). The 8‐repetition maximum (RM) strength of unilateral knee extension (71 ± 12.0 kg) and unilateral 45° leg press (299 ± 44.8 kg) for both legs was assessed 4–5 days prior to the first experimental exercise bout. At the same time, familiarization for the single‐leg squats and walking lunges was performed. On the day of each trial, the resistance exercise bout included six sets of 45° leg press and knee extensions (8,8,10,12,10, and 10 RM), and three sets of single‐leg squats and walking lunges (12 repetitions in each set).

For the CWI trial, 5 min after exercise, the participants sat in an inflatable bath (iCool iBody, Miami, Australia) for 10 min. The bath contained water that was maintained at 10 ± 0.3°C, and the participants were immersed to the level of their waist. For the control active recovery (ACT; light recovery exercise performed at room temperature) trial, instead of undergoing CWI the participants exercised at room temperature on a stationary bicycle ergometer (Wattbike, Nottingham, UK) at a light, self‐selected intensity (59.5 ± 9.4 W) for 10 min. Participants were prevented from showering or bathing for a further 2 h. The order of the CWI and ACT trials was randomized and counterbalanced.

Participants consumed their habitual diet, excluding alcohol, for the 48 h before trial 1, which included their preferred pre‐exercise meal of choice 2 h before the pre‐exercise biopsy. They were provided with and consumed 30 g of whey protein (Body Science, Gold Coast, Australia) at the end of exercise, before CWI and ACT, after which they fasted until the 2 h biopsy. Following this, another 30 g of the same whey protein was consumed. Participants consumed a habitual diet, excluding alcohol, between the 2 and 48‐h biopsies. Subjects were asked to refrain from strenuous activity over the 48 h before trial 1, and between the 2 and 48‐h biopsies. All activity and diet were recorded by participants in diaries relating to trial one, and then replicated for trial 2.

Muscle biopsies were collected from the vastus lateralis before, 2, 24, and 48 h post‐exercise and immediately snap frozen in liquid nitrogen and stored at −80°C until further analysis. All participants were informed of the requirements and potential risks of the studies prior to providing their written informed consent. The experimental procedures adhered to the standards set by the latest version of the Declaration of Helsinki and were approved by the Human Research Ethics Committee of The University of Queensland (2013001426).

### 2.1 Immunoblotting

Muscle tissue (~25 mg) was homogenized in ice‐cold RIPA lysis buffer (Millipore, Temecula, CA, USA) supplemented with the Halt™ protease and phosphatase inhibitor cocktail (Thermo Scientific). After centrifugation 15,000 *g* for 10 min at 4°C, supernatants were collected, and total protein concentrations were determined using the Pierce™ BCA Protein Assay Kit (Thermo Scientific). Equal amounts of protein were boiled in Laemmli buffer at 95°C for 5 min. Twenty micrograms of protein was separated by SDS‐PAGE and transferred to PVDF membranes (Bio‐Rad Laboratories, Inc., CA) using the semidry Trans‐Blot Turbo™ device (Bio‐Rad). Membranes were incubated overnight with primary antibodies (Abcam) against Drosha (ab85027), Exportin‐5 (ab129006), Dicer (ab13502), VEGF (ab46154), SPRED‐1 (ab77079), and EGF1 (ab9695). Antibodies were used at 1:1000 dilution, except VEGF and SPRED1, which were used at 1:2000 and 1:500, respectively. The following day membranes were incubated for 1 h at room temperature with appropriate anti‐rabbit or anti‐mouse horseradish peroxidase (HRP) conjugated secondary antibodies (Jackson ImmunoResearch Laboratories, PA) (1:10,000). The membranes were exposed on a ChemiDoc image device (Bio‐Rad) using enhanced chemiluminescence reagent (ECL Select kit; GE Healthcare Ltd.). Bands were quantified using ImageJ software (NIH, Bethesda, MD). Western blot data were normalized to the housekeeping protein GAPDH (Abcam, ab9485) (1: 10,000).

### 2.2 RNA extraction and quantitative real‐time PCR

Total RNA was extracted from 20 mg of muscle tissue using the AllPrep® DNA/RNA/miRNA Universal Kit (QIAGEN GmbH). 1500 ng of input RNA were then used for cDNA synthesis using High‐Capacity RNA‐to‐cDNA™ kit (Life Technologies). Messenger RNA (mRNA) was measured by RT‐PCR on a LightCycler 480 II (Roche Applied Science) using SYBR Green I Master Mix (Roche Applied Science). Target mRNAs were *DROSHA*, *DICER*, *EXPORTIN‐5*, *NCAM*, *PAX7*, *VEGF*, *and SPRED‐1*. Primers were designed using BLAST software (Table 1). Relative fold changes were determined using the 2^−ΔΔCT^ method (Schmittgen & Livak, 2008). The geometric mean of three reference genes was used for normalization (Vandesompele et al., 2002). The human reference genes (Eisenberg & Levanon, 2013) chromosome 1 open reading frame 43 (*C1orf43*), charged multivesicular body protein 2A (*CHMP2A*) and ER membrane protein complex subunit 7 (*EMC7*) were identified as the least variable, and therefore used as reference genes (Table 1).

**TABLE 1:** mRNA sequences. Forward and reverse sequences of analyzed genes.

Gene	Sequence
*DROSHA (Forward)*	CGATCCAGGCCAGATGAATGA
*DROSHA (Reverse)*	TAGGACGACAGGGCTTGATG
*EXPORTIN‐5 (Forward)*	ATCCTGTCCTGCGTCCTTAC
*EXPORTIN‐5 (Reverse)*	TCTGGGGGCCTTACTTTCTTC
*DICER (Forward)*	AAAATTGGCGAACTGGATGACC
*DICER (Reverse)*	TTTGGGTAGCACTGCCTTCG
*NCAM 1 (Forward)*	GCAGCGAAGAAAAGACTCTGG
*NCAM 1 (Reverse)*	GCAGATGTACTCTCCGGCAT
*PAX7 (Forward)*	CCTTTGGAAGTGTCCACCCC
*PAX7 (Reverse)*	TCGCCCATTGATGAAGACCC
*VEGF (Forward)*	TCTTCAAGCCATCCTGTGT
*VEGF (Reverse)*	CTTTCTTTGGTCTGCATTC
*SPRED‐1 (Forward)*	CGTTTCAAAGTCCTGCTGATG
*SPRED‐1 (Reverse)*	CATTTGCTTGTAAGTCATCTGCCC
*C1ORF43 (Forward)*	CTATGGGACAGGGGTCTTTGG
*C1ORF43 (Forward)*	TTTGGCTGCTGACTGGTGAT
*CHMP2A (Forward)*	CGCTATGTGCGCAAGTTTGT
*CHMP2A (Forward)*	GGGGCAACTTCAGCTGTCTG
*EMC7 (Forward)*	GGGCTGGACAGACTTTCTAATG
*EMC7 (Reverse)*	CTCCATTTCCCGTCTCATGTCAG

### 2.3 miRNA reverse transcription and PCR

Ten nanograms of RNA from muscle tissue was used for cDNA synthesis using TaqMan™ Advanced miRNA cDNA Synthesis Kit (Thermo Fisher Scientific) and miRNA abundances were measured by RT‐PCR on a QuantStudio 6 instrument (Thermo Fisher Scientific) using Applied Biosystems Fast Advanced Master Mix (Thermo Fisher Scientific).

Target miRNAs were chosen from a literature search of those previously implicated in the regulation of satellite cell dynamics and angiogenesis (Table 2). The geometric mean of three miRNAs were used for normalization, using miRNAs that showed the least variance in the current sample set (miR‐191, ‐361 and ‐320a). Data were analyzed using the 2^−ΔΔCT^ method (Schmittgen & Livak, 2008).

**TABLE 2:** miRs analyzed. Classification and identification number.

miR	ID number
hsa‐miR‐126‐3p	477887_mir
hsa‐miR‐133a‐3p	478511_mir
hsa‐miR‐1‐3p	477820_mir
hsa‐miR‐15a‐5p	477858_mir
hsa‐miR‐361‐5p	478056_mir
hsa‐miR‐320a	478594_mir
hsa‐miR‐191‐5p	477952_mir

### 2.4 Statistical analyses

All data were tested for normality using Shapiro–Wilk and equal variance using Levene's Test. Where appropriate, data were log transformed and normality and equal variance were restored. These log transformed data were used to perform statistical analysis. Raw data are presented in the figures. All data were analyzed with SigmaPlot software (Windows v. 12.1, Systat 218 Software Inc.) using two‐way repeated measures ANOVA, with time as a within‐subject factor and trial as a between‐subject factor. Where time × trial interactions were observed, pair‐wise differences from pre versus post‐exercise within and between the ACT and CWI trials were assessed with Holm‐Sidak post hoc tests. Statistical significance was accepted at *p* < 0.05.

## 3 RESULTS

### 3.1 Regulators of miRNA biogenesis and maturation

**FIGURE 1:**
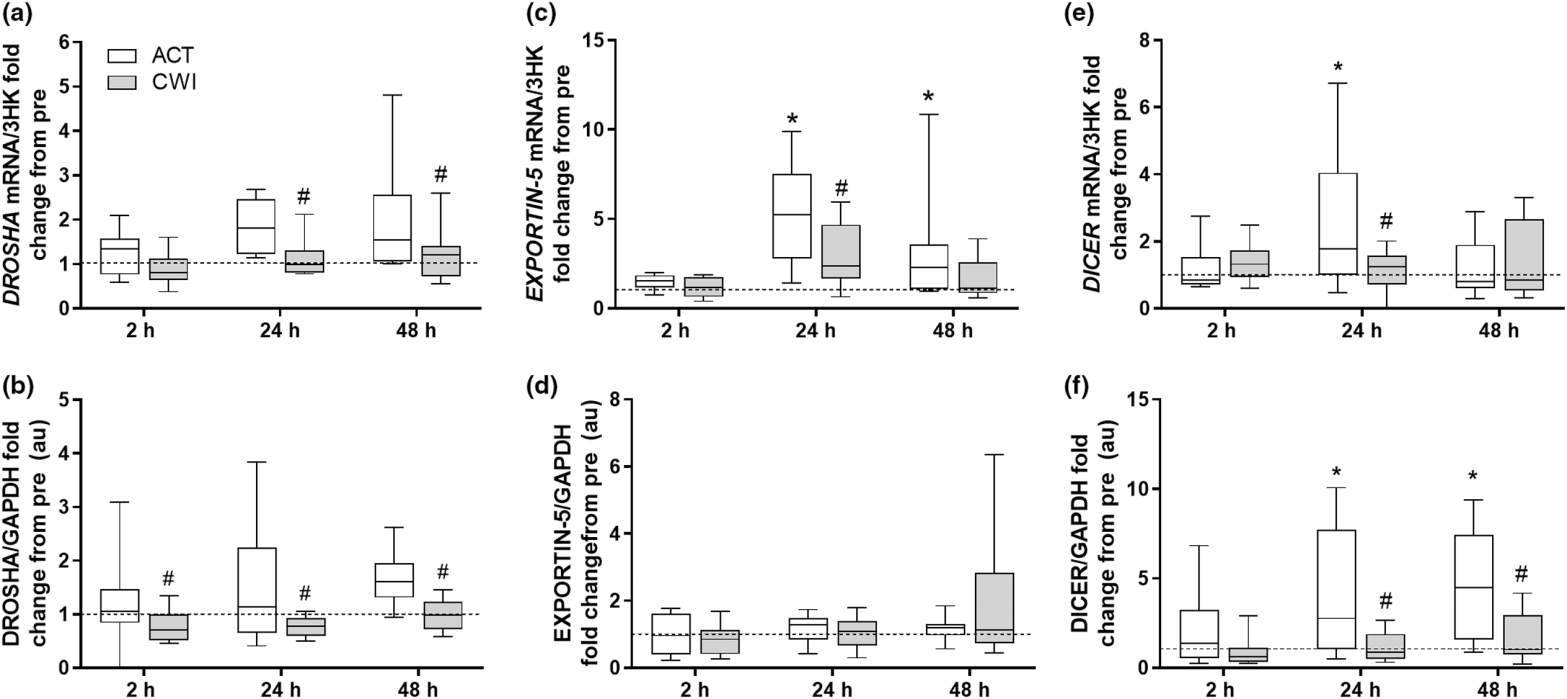
Effect of CWI on components regulating microRNA biogenesis. Fold change relative to respective pre‐exercise (a, b) *DROSHA mR*NA and protein, (c, d) *EXPORTIN‐5 mRNA* and protein, and **(**E, F ***)*** *DICER mRNA* and protein, respectively. *p < 0.*05* respective pre‐exercise values; # *p < 0*.*05* difference between recovery strategies at the respective time point. Data are expressed as box and whisker plots with spread plotted as 95% CI. The horizontal dotted line reflects pre‐exercise values.

*DROSHA* (trial × time interaction *p* = 0.045) mRNA expression was reduced after the CWI trial, compared with the ACT trial, when measured in the biopsied muscle samples 24, and 48 h following the completion of the exercise (*p* = 0.025 and *p* = 0.017, respectively) (Figure 1a). Drosha protein abundances remained unaltered after the CWI trial yet were significantly lower than after the ACT trial at all timepoints (2, 24, and 48 h; *p* = 0.039, *p* = 0.033, and *p* = 0.014, respectively) (Figure 1b).

*EXPORTIN*‐5 (trial × time interaction *p* = 0.047) mRNA increased at 24 and 48 h post‐exercise in the ACT trial only. Additionally, *EXPORTIN*‐5 was lower at 24 h after the CWI trial, compared with the ACT trial (*p* = 0.008) (Figure 1c). Protein expression of EXPORTIN‐5 was unresponsive to either exercise or recovery modality (trial × time interaction *p* = 0.416) (Figure 1d).

The mRNA of the *DICER* showed a trial × time interaction (*p* = 0.034) (Figure 1e). At 24 h following exercise, it was attenuated in the CWI trial compared to the ACT trial (*p* = 0.004). At 24 h *DICER* mRNA was also elevated compared to pre‐exercise levels only in the ACT group (*p* = 0.007). The protein expression of DICER (Figure 1f) also showed an interaction (trial × time *p* = 0.031) and was attenuated in the CWI trial compared to the ACT trial at both 24 and 48 h (*p* = 0.002 and *p* = 0.003), respectively. DICER protein in the ACT trial was also elevated at 24 and 48 h compared with rest (*p* = 0.002 and *p* < 0.001), respectively.

### 3.2 miRNA regulators of myogenesis

**FIGURE 2:**
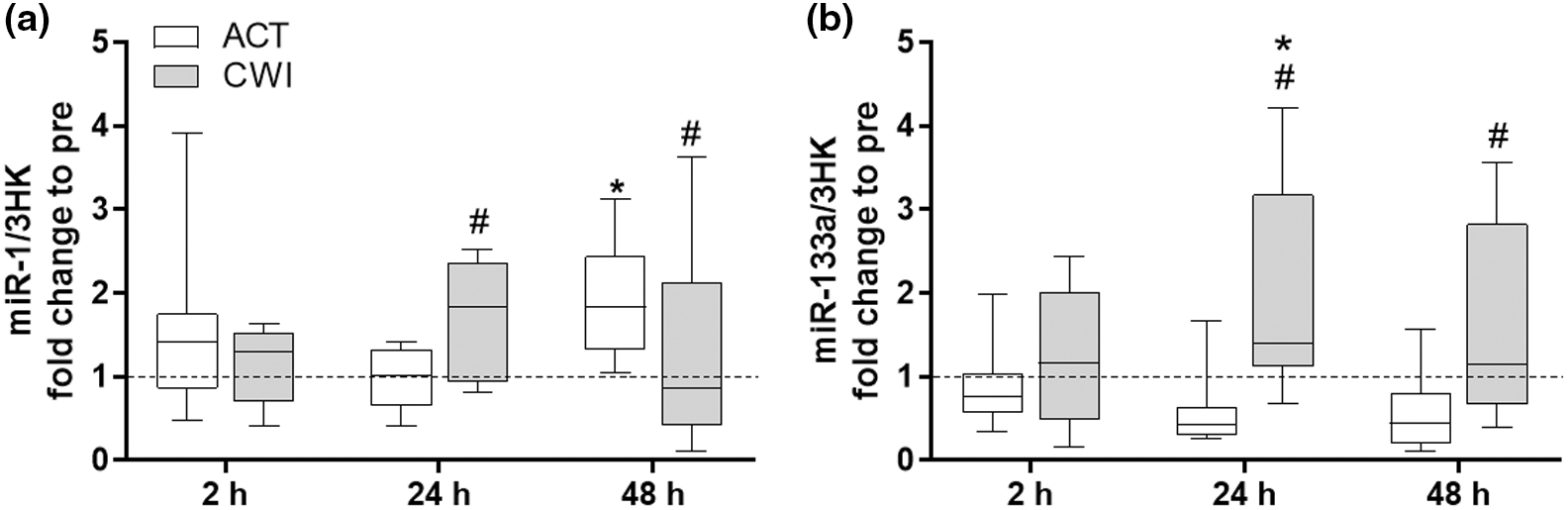
Effect of CWI on miRNAs that regulate myogenesis. Fold change relative to respective pre‐exercise. (a) miR‐1 and (b) miR‐133a, respectively. *p < 0.*05* respective pre‐exercise values; # *p < 0*.*05* difference between recovery strategies at the respective time point. Data are expressed as box and whisker plots with spread plotted as 95% CI. The horizontal dotted line reflects pre‐exercise values.

miR‐1 and ‐133a are reported inhibitors of myogenic cell differentiation and proliferation. Both miRNAs showed trial × time interactions (*p* = 0.008 and 0.005, respectively) (Figure 2a,b). At 24 h there was an increase in miR‐1 after the CWI trial compared with the ACT trial (*p* = 0.023), while at 48 h this pattern was reversed, with a lower miR‐1 expression after in the CWI trial compared to the ACT trial (*p* = 0.04). Expression of miR‐1 was also increased at 48 h compared to pre‐exercise levels in the ACT trial alone (*p* = 0.012. miR‐133a showed an increased expression at both 24 and 48 h after the CWI trial compared with the ACT trial (*p* < 0.001 and *p* = 0.007), respectively. In the CWI trial, miR‐133a was also elevated at 24 h following exercise when compared with pre‐exercise levels (*p* = 0.008).

### 3.3 miRNA regulators of angiogenic signaling

**FIGURE 3:**
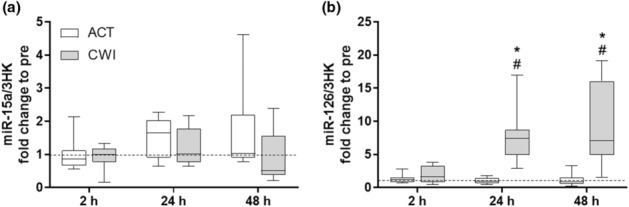
Effect of CWI on miRNAs that regulate angiogenesis. Fold change relative to respective pre‐exercise. (a) miR‐15a and (b) miR‐126, respectively. *p < 0.*05* respective pre‐exercise values; # *p < 0*.*05* difference between recovery strategies at the respective time point. Data are expressed as box and whisker plots with spread plotted as 95% CI. The horizontal dotted line reflects pre‐exercise values.

miR‐15a (a negative regulator of VEGF expression) was also not altered after exercise or CWI (trial × time interaction *p* = 0.215) (Figure 3a). miR‐126 showed a trial × time interaction *p* < 0.001 (Figure 3b). Expression of miR‐126 was elevated only in the CWI trial at both 24 and 48 h compared with after the ACT trial (*p* < 0.001 and *p* < 0.001, respectively) and pre‐exercise levels (*p* < 0.001 and *p* < 0.001, respectively).

### 3.4 Satellite cell gene expression

**FIGURE 4:**
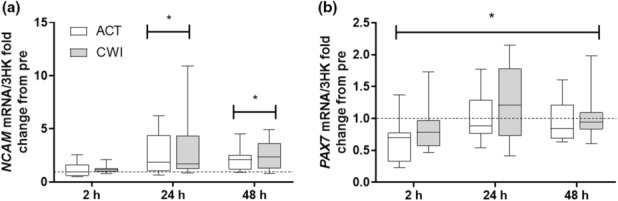
Effect of CWI on satellite cell dependent myogenesis. Fold change relative to respective pre‐exercise. (a) *NCAM mRNA* and (b) *PAX7 mRNA*. *p < 0.*05* respective pre‐exercise values; # *p < 0*.*05* difference between recovery strategies at the respective time point. Data are expressed as box and whisker plots with spread plotted as 95% CI. The horizontal dotted line reflects pre‐exercise values.

*NCAM* mRNA and *PAX7* mRNA showed time main effects following exercise (*p* < 0.001 and *p* = 0.01, respectively) (Figure 4a,b). *NCAM* mRNA was significantly elevated at 24 and 48 h and was not regulated by recovery modality (*p* < 0.001 and *p* = 0.021, respectively). *PAX7* mRNA was significantly elevated at 2, 24, and 48 h and was not regulated by recovery modality.

### 3.5 Angiogenic gene and protein expression

**FIGURE 5:**
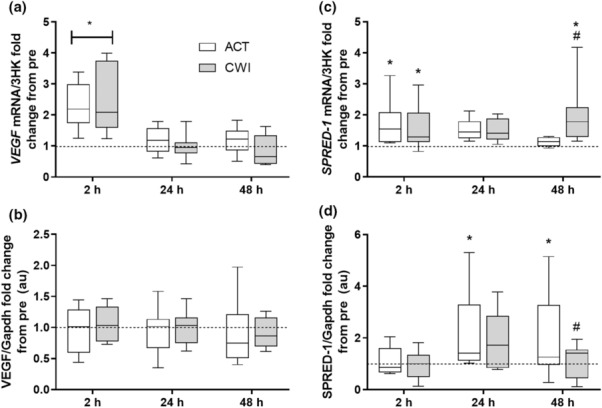
Effect of CWI on angiogenesis markers. Fold change relative to respective pre‐exercise. (a, b) *VEGF mRNA* and protein, and (c, d) *SPRED‐1 mRNA* and protein. *p < 0.*05* respective pre‐exercise values; # *p < 0*.*05* difference between recovery strategies at the respective time point. Data are expressed as box and whisker plots with spread plotted as 95% CI. The horizontal dotted line reflects pre‐exercise values.

*VEGF* mRNA demonstrated a main time effect *p* < 0.001 with a specific increase at 2 h following exercise in both trials (*p* < 0.001) (Figure 5a). However, VEGF protein was not altered by exercise or CWI (trial × time interaction *p* = 0.906) (Figure 5b). The inhibitor of angiogenesis, *SPRED‐1* mRNA (trial × time interaction *p* = 0.027) was elevated above pre‐exercise in both the CWI and ACT trials at 2 h following exercise (*p* = 0.021 and *p* = 0.047, respectively) (Figure 5c). At 48 h there was an increased *SPRED‐1* mRNA expression in the CWI trial compared with both the ACT trial and respective pre‐exercise values (*p* = 0.003 and *p* < 0.001). Expression of SPRED‐1 protein (trial × time interaction *p* = 0.037) was increased from pre‐exercise values in the ACT trial only at both 24 and 48 h (*p* = 0.002 and *p* = 0.035) (Figure 5d). Additionally, a significant attenuation in SPRED‐1 protein expression was evident at 48 h after the CWI trial compared with after the ACT trial (*p* = 0.039).

**FIGURE 6:**
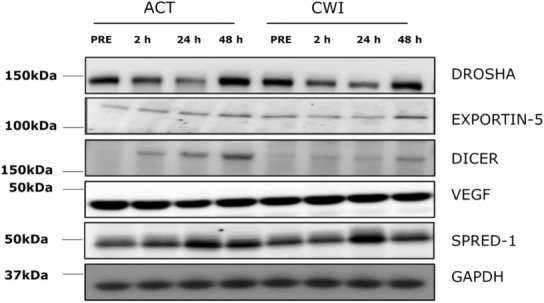
Representative immunoblot images.

Representative blots of analyzed proteins are shown in Figure 6.

## 4 DISCUSSION

The current study investigated the mechanisms regulating skeletal muscle miRNAs following a single bout of leg resistance exercise, followed by either CWI or ACT. It was demonstrated that brief CWI exposure has a small yet persistent impact to suppress exercise‐induced increases in gene and protein abundances of DROSHA, EXPORTIN‐5, and DICER. Therefore, our findings are contrary to our hypothesis that CWI would transiently increase muscle levels of DICER, given the previous evidence for the temperature sensitivity of this protein (Pilotte et al., 2011; Russell et al., 2013). This study further examined whether the altered responsiveness of these major components of miRNA processing to CWI exerted an impact on the measured abundances of several key myogenic miRNA species. There was a divergence in the abundance of miRNA‐1, miRNA‐133‐a, and miRNA‐126, with a tendency for greater expression after the CWI trial. Nevertheless, further potential targets for the actions of these miRNAs, including *NCAM* and *PAX7* (targets of miRNA 1 and ‐133‐a), as well as VEGF and SPRED‐1 (targets of miRNA‐15a and ‐126), were not altered by CWI.

There has been limited analysis of the responsiveness of the pathway responsible for miRNA biogenesis, transport, and maturation to acute exercise stress and post‐exercise recovery modalities. In untrained individuals, a single bout of cycle ergometry alone, or with an additional bout of knee extensor resistance exercise, was found to increase muscle mRNA expression of *DROSHA*, *EXPORTIN‐5*, and *DICER* at 1 and 3 h post‐exercise (Garner et al., 2020; Russell et al., 2013). Additionally, following 10 days of endurance training, the initial increase in expression observed 3 h following the final acute exercise bout was attenuated, relative to the activation following the first (untrained) exercise bout (Russell et al., 2013). Consistent with these prior studies, we report that miRNA biosynthetic pathways are induced in human muscle at 24 and 48 h of recovery from an acute bout of resistance exercise (although we found no change at 2 h). We chose to focus on these later time‐points so that we could also identify corresponding changes in protein expression. Indeed, to our knowledge the current study is the first to report on the protein level modulation of these important miRNA regulatory factors in human skeletal muscle. In this regard, our data indicate a substantial increase in intramuscular DICER protein at 24 and 48 h post‐exercise. The most striking finding in the current study is that CWI almost completely abolished all exercise‐induced changes in *DROSHA*, *EXPORTIN‐5*, and *DICER*. Precisely how this may impact upon muscle adaptations to exercise is unclear and warrants further investigation.

DROSHA or DICER knockout (KO) mice were recently shown to display significant alterations in muscle miRNA abundances (Garner et al., 2020; Vechetti Jr et al., 2019). Despite this, these KO mice did not differ obviously from wild type controls with regard to muscle phenotype (Vechetti Jr et al., 2019) or endurance performance (Oikawa et al., 2019). Therefore in the current study, analysis of key myoMiRs (muscle‐specific miRNAs) was also made (McCarthy & Esser, 2007). Of the analyzed miRNA species, we included miR‐1 and ‐133a, because they have previously been identified as inhibitors of satellite cell activity and muscle protein synthesis (Drummond et al., 2008; D'Souza, Zeng, Markworth, et al., 2019). From the current study, it is apparent that miR‐1 and ‐133a expression were elevated after the CWI compared to ACT. This new finding may help explain the deleterious effects of CWI on resistance exercise‐induced muscle anabolic signaling, protein synthesis, and hypertrophy previously reported by us and others (Figueiredo et al., 2016; Roberts et al., 2015). miR‐1/133 targets several pro‐growth genes and has been shown to be downregulated during muscle hypertrophy in mice (McCarthy & Esser, 2007). On this basis of prior published work showing that CWI can stimulate muscle angiogenesis (D'Souza, Zeng, Markworth, et al., 2018) analysis was included for miR‐15a and miR‐126, which are reported to be important regulators of angiogenesis‐related pathways. No change was seen in the anti‐angiogenic miR‐15a, but the abundance of pro‐angiogenic miR‐126 was markedly elevated from pre‐exercise levels at 24 and 48 h following exercise in the CWI trial when compared to ACT.

Activation and the subsequent proliferation, differentiation, and fusion of satellite cells following resistance exercise is thought to be central in myofiber repair following resistance exercise. *NCAM* and *PAX7* mRNA is typically upregulated in human muscle biopsies within hours of exercise, and remains upregulated for up to 24 h following exercise (Nederveen et al., 2017). In this study, we observed a small increase in *NCAM* and *PAX7* mRNA following exercise. The absence of differences between the CWI and ACT trials at 24 and 48 h suggests no effect of CWI on muscle expression of these myogenic factors. However, when paired with the observed miRNA changes, CWI and ACT may negatively influence the translation of target *NCAM* and *PAX7* mRNA species. These data are consistent with our previous observations of abolished increase in Pax7 positive cells for up to 48 h following CWI treatment, when compared to ACT after intense resistance exercise (D'Souza, Bjørnsen, et al., 2017). This in turn may contribute to the impaired muscle adaptation to training, as previously reported with CWI (D'Souza, Zeng, Markworth, et al., 2018; Roberts et al., 2015).

We have previously identified that CWI recovery across a period of resistance training results in a noticeable increase in muscle angiogenesis (D'Souza, Zeng, Markworth, et al., 2018). Although we observed that the proangiogenic *VEGF* mRNA rapidly increased following exercise, consistent with previous studies (Gavin et al., 2007; Hoier & Hellsten, 2014), we did observe corresponding changes in VEGF protein content. The anti‐angiogenic factor *SPRED‐1* mRNA demonstrated a modest (less than 2‐fold) increase, also without marked changes in its protein abundance. The robust upregulation of miR‐126 after CWI versus ACT at 24 and 48 h may contribute to the observed reduction in SPRED‐1 protein content at 48 h post‐CWI (D'Souza, Zeng, Markworth, et al., 2018; Meng et al., 2012; Mitchell et al., 2018; Wang et al., 2008; Wang et al., 2016). Therefore, it is possible that a reduction in the inhibitory actions of SPRED‐1 may contribute to the greater angiogenesis following repeated exercise and CWI, as previously reported. However, this requires careful validation given that we also demonstrate a possibility for suppressed inhibition of angiogenesis, through downregulated SPRED‐1 gene expression.

The pattern of gene and protein expression for either myogenesis or angiogenesis, including the abundance of key miRNA species fail to adequately explain the molecular response to CWI, and how these might affect the resultant phenotype adaptations. It is of interest to note the measured increased abundance of miR‐133a and ‐126 at 2 h post CWI contrast with the suppressive impacts on the miRNA processing genes. Without detailed analysis inclusive of greater time points, measurements of miRNA synthesis rates, and more comprehensive analysis of a wider array of miRNA species any conclusions can only be speculative. There is indeed the possibility that the are differing processing mechanisms in the maturation of classes or groups of miRNAs (Vechetti Jr et al., 2019). Furthermore, miRNAs are thought to function as distance signaling molecules and transported via extracellular vesicles (D'Souza, Woodhead, Zeng, et al., 2018; Ji & Guo, 2019). Hence, whether these measured miRNAs were indeed solely synthesized within the sampled biopsied tissue was not elucidated. Future studies, potentially in‐vitro with the use of cultured myoblasts might be a strategy to delineate the independent impact of cold stimuli more comprehensively on temperature sensitive genes and miRNA.

An important feature of the current study is that the analysis of miRNA abundance was performed using probes that cannot differentiate immature from mature miRNA forms. Critically, small RNA sequencing will need to be applied in future studies, not only to investigate the abundances of miRNA isoforms, but also to provide the opportunity to assess the global changes in all non‐coded small RNA species. The current study demonstrate that CWI blocks the increase in DROSHA and DICER protein levels following resistance exercise. How this affects the cellular concentrations of mature miRNA and the resultant functions remains unclear. Our results did not provide any specific molecular signature that was indicative of the phenotype adaptations of CWI in response to training. Apart from the rapid (2 h) increased in *VEGF* mRNA expression, our results show little evidence for any consistent trend that might indicate a transcriptional mechanism of our previously reported increased angiogenic response to CWI. However, our findings that CWI following resistance exercise promote expression of miR‐1/133 may help explain how CWI blunts the anabolic signaling and muscle protein synthesis in response to resistance exercise.

## AUTHOR CONTRIBUTIONS

The study was designed by Randall F. D'Souza, Llion A Roberts, James F. Markworth, Truls Raastad, Jeff S. Coombes, Jonathan M. Peake, David Cameron‐Smith, Cameron J. Mitchell. Performed experiments: Randall F. D'Souza, Vandre C. Figueiredo, James F. Markworth, Nina Zeng, and Christopher P. Hedges. Sample Collection: Llion A Roberts and Jonathan M. Peake. Analyzed data: Randall F. D'Souza, James F. Markworth, Christopher P. Hedges. Drafted manuscript: Randall F. D'Souza. All authors critically evaluated and have approved the final content of the manuscript. Randall F. D'Souza is responsible for the final content of the manuscript.

## FUNDING INFORMATION

This study was funded by research grants from the American College of Sport Medicine (ACSM) Research Foundation and Exercise and Sport Science Australia (ESSA) awarded to LR and JP, and a grant from Queensland University of Technology awarded to JP. LR was supported by an International Postgraduate Research Scholarship from the University of Queensland. The laboratory analysis was funded by core funding from AgResearch Limited (contracts A19079 and A21246) to DC‐S. The authors acknowledge the support of Body Science Nutrition (Gold Coast, Australia) for the provision of the nutritional supplement.
